# The Hospital. Nursing Section

**Published:** 1903-02-28

**Authors:** 


### The Hospital.

### Nursing Section.

Contributions for this Section of "The Hospital" should be addressed to the Editor, "The Hospital" Nursing Section, 28 & 29 Southampton Street, Strand, London, W.C.

No. 857.—Vol. XXXIII. *SATURDAY, FEBRUARY* 28, 1903.

### Notes on News from the Nursing World.

### THE NURSING STAFF AT THE HERBERT HOSPITAL.

The matron-in-chief of Queen Alexandra's Imperial Military Nursing Service sends us a complete list of the appointments to the Herbert Hospital at Woolwich. As already announced Miss Beatrice Jones, who was trained at St. Bartholomew's Hospital, is the matron. There are seven sisters, namely, Miss W. Potter, Miss G. A. Magill, Miss D. V. Briscoe, who previously belonged to the Army Nursing Service; Miss G. E. Larner, who was trained at St. Bartholomew's Hospital; Miss S. Lemming, who was trained at the Queen's Hospital, Birmingham; Miss E. C. Cheetham, who was trained at Guy's Hospital; and Miss L. M. Lyall, who was trained at the Metropolitan Hospital. The staff nurses are twelve in number, and consist of Miss C. C. R. Moir, who was trained at the London Homœopathic Hospital; Miss A. A. Wilson, and Miss E. J. M. Keene, who were trained at St. Bartholomew's Hospital; Miss E. M. Pettle, who was trained at Croydon Infirmary; Miss E. C. Humphreys, and Miss A. M. Fitzgerald, who were trained at the London Hospital; Miss M. Kendall, Miss S. R. Hughes-Hallett, and Miss L. A. Rideout, who were trained at Addenbrooke's Hospital, Cambridge; Miss F. E. C. Watson, who was trained at Nottingham General Hospital; Miss E. M. Bickerdike, who was trained at Blackburn Infirmary; and Miss M. Pedler, who was trained at University College Hospital, London. Several members of the staff, including the matron and Sister Cheetham, have had considerable experience of nursing in military hospitals during the South African war, as members of the Army Nursing Service Reserve.

### NURSING ASSISTANT.

The Hospital Committee of the Bristol Board of Guardians have pronounced strongly against the proposal to create an order of qualified nurses, which in common with other people, they consider would lead to misunderstanding. They think that the term "qualified nurse" should only be applied to persons who have completed the three years' course of training, and they suggest that in respect to others the designation of "nursing assistant" should be used. This, they are of opinion, would make it clear that the individual holding the certificate of a minor school had only received partial training and was not fully competent to act except under trained supervision. The recommendation of the Bristol Board is the most sensible alternative which has yet been put forward. The term "nursing assistant" has certainly the advantage of being incapable of misunderstanding.

### DR. RHODES AND THE MATRONS.

As the postscript of an epistolary attack on Miss Wilson and the Workhouse Nursing Association, Dr. Milson Rhodes protests against the Association sending out "their circulars signed by the Matron of X. or Y. unions." He adds: "These ladies have a perfect right to sign their names as Angelina Gushington or Lydia Languish, but they have no more right to sign as the Matron of X. than the Clerk of X. has without consulting the Board, whose officers they are." Dr. Rhodes misrepresents the nature of the document to which he refers. The Workhouse Nursing Association have not "sent out circulars." They sent out copies of the memorial addressed to the President of the Local Government Board against the creation of that ridiculous personage the qualified nurse of one year's training, with the names of all the matrons of the principal workhouse infirmaries appended to it. We conclude that Dr. Rhodes would like to deny to the matrons the right to express their opinion on subjects to which the lay public look to them for guidance because they have the temerity to differ from him, and have helped to make it clear to Mr. Walter Long that the foremost members of the nursing profession are completely hostile to the recommendation of the Departmental Committee which Dr. Rhodes endorses.

### VICTORIAN TRAINED NURSES' ASSOCIATION.

The first examination under the auspices of the Victorian Trained Nurses' Association was held in December at Melbourne by the conjoint board of examiners, which is constituted by the senior lecturer of the Melbourne, the Alfred, and the Children's Hospitals, the matrons of the Melbourne and of the Alfred Hospitals. The candidates were required to write a paper on medical nursing and a second on surgical nursing, and there was also an oral examination on each subject. Thirty-five candidates presented themselves, of whom 12 were from Melbourne Hospital, 10 from the Children's Hospital, Melbourne, and the remainder from various institutions. From Ballarat and Bendigo reports were received stating that the practical work of all the candidates reached the pass standard; and the papers were all judged of sufficient merit to pass.

### THE WAR OFFICE AND THE "WEAKER VESSEL."

A brief, but sufficient, reply is made in our issue of to-day by the Reserve Sister whose article on "Nursing by Orderlies" provoked the suggestive criticism which we recently published from Private Power. With regard to the remarks of the orderly at Kroonstad as to women being the weaker vessel, a Reserve Sister makes the telling retort that this is not the view of the War Office on the subject, because the War Office expects the sister to control the orderlies. She might have added that the essential difference in the eyes of the War Office, as shown by the regulations of Queen Alexandra's Imperial Military Nursing Service is that the sister holds the commission which the orderlies, as represented by Private Power, consider impossible, and is entitled by rank, as well as by courtesy, to the salute, which on the same authority will never be given by the "stronger vessel."

### MEETING OF THE SHAM CO-OPERATION AT GLASGOW.

THE special general meeting of the Glasgow and West of Scotland Co-operation of Trained Nurses on Monday appears to have been of a similar character to the scheme of the executive. Mr. John Stewart Bannatyne, of Kelvinside, Glasgow, writes to inform us that in his capacity as a member of the Co-operation he attended on Monday at the rooms of the Religious Institution, where, it was announced, the special general meeting would be held, in order to adopt the new constitution, but he was met at the door by the Co-operation solicitor, who, without stating any reason for his attitude, refused him admission. Mr. Bannatyne, who is himself a solicitor, produced an admission ticket in vain, and he now requests the solicitor to the Co-operation to explain through our columns why he usurped the duties of commissionaire. It is not an unreasonable inference on the part of Mr. Bannatyne that the fact that at the annual meeting he was instrumental in preventing the new constitution which the solicitor had framed from being adopted, may account for that official's evident anxiety to exclude him from the exercise of his legal rights. Of course, the questions which Mr. Bannatyne intended to address to the executive regarding the written apology which the nurses belonging to the Co-operation were recently compelled to make, could not in the circumstances be put, but the exclusion of members of the organisation from the meeting reduced the proceedings to a farce.

### RIVAL ASSOCIATIONS IN CUMBERLAND.

ON behalf of the Ireby, Uldale, and Belton Nursing Associations a very successful sale of work has just been held. The sum realised was a little over £50, and it is a feature in connection with this Association that the working classes of the district, by whom it is materially supported, insist upon a fully trained nurse being employed. Although the population is under a thousand, the Cumberland Nursing Association have also a nurse of six months' training in the district, who works under the auspices of a local committee with Lady Gillford as head. Persistence in the opposition causes a good deal of ill feeling. We are glad to learn, however, that the Deputy Speaker of the House of Commons has sent the association supported by the working classes a cheque for £2 towards their efforts, and no doubt other outside help will be forthcoming as time goes on.

### THE QUESTION OF SUNDAY VISITING.

IT is proposed that visiting should be allowed at the Edmonton Union Infirmary on Sunday afternoons. Objection is offered on the ground that the Infirmary would thus be made a promenade for the crowd on Sunday, and that all the nurses would in consequence have to be on duty from two till six o'clock. But visitors are allowed at general hospitals on Sundays, and no hardship is inflicted upon anyone. Edmonton too is one of the districts largely inhabitated by persons who can only visit the Infirmary on Sunday because they are at work all through the week in London. If the concession to the public involves extra duty to the nurses, it is quite possible to compensate them by allowing a little more time off during the week. We do not believe that any nursing staff would desire to stand in the way of enabling the sufferers in the wards to see their friends on Sunday.

### A COMPLIMENT TO THE COUNTESS OF MEATH.

THE Guardians of the Oxford Incorporation, at their last meeting, passed a resolution recognising the great usefulness of the Meath Workhouse Nursing Association, "which has supplied them with a number of efficient nurses during the past few years." The Guardians, "also considering the immense benefit which has accrued to many of the inmates of the workhouse from the adoption of the Brabazon Employment Scheme," have placed on record "their deep sense of the valuable services which the right honourable the Countess of Meath has rendered to the poor in connection with both these institutions," and have expressed to her their sincere thanks.

### NOTTS NURSING FEDERATION.

PRESIDING at the annual meeting of the Notts Nursing Federation at Nottingham on Monday, Earl Manvers pointed out how the poor in their homes are gradually getting more and more to appreciate the great advantage of skilled nursing, against which some years ago they entertained rather a prejudice. This was being gradually overcome, and from his experience of the work in various districts of the county he felt sure the people were coming to recognise that skilful nursing saved an enormous amount of unnecessary suffering, and that an organisation such as that Federation performed a considerable amount of useful work. The Hon. and Rev. A. E. Bertie also spoke of the prejudice which existed in former days against skilled nursing, and mentioned the case of the first nurse who came to his district. For a time her path was by no means of a smooth character, but after a few years the prejudice was entirely removed, and now they had to have two nurses.

### CYCLING ON THE FOOTPATH.

A DISTRICT nurse was fined this month for cycling on the footpath, and she writes to us to explain the circumstances of the case. She had to report to the doctor the alarming condition of a patient—a maternity case—and get to another woman, who was dying, and has since died, at the other end of her district, as soon as possible. The roads, she states, were so dirty that she could not make progress on her cycle, and accordingly she ran off on to the side path. Her reasons for this admitted act of trespass were explained to the police constable, and afterwards to the magistrate, by both the doctor and herself, but with no avail, and she suggests the introduction of a clause in the Act of Parliament "to protect the medical profession and nurses from impositions for doing their duty." It would scarcely be wise or practicable to make the exceptions she suggests, but magistrates can always exercise their discretion. In this instance they might fitly have discharged the offender with a caution.

### DISTRICT AND PRIVATE NURSING AT TORQUAY.

The committee of the Torquay Nurses' Institution, which has existed 25 years, have determined to discontinue private nursing, and for the future to devote attention only to district nursing. They have been unable to ignore the fact that some of the best friends of the institution have not regarded with favour the union of professionalism and philanthropy, and the majority believe that the abolition of the commercial side—the private nursing—will have the effect of securing a larger measure of support. An additional £150 a year will be required for carrying on the district nursing among the poor, to whom no charge whatever is made. The work of the four district nurses is spoken of in the highest terms of praise. Last year they paid 13,725 visits to 326 patients. Miss Thomson having resigned the position of lady superintendent, her successor will be Miss Robinson. Now that the Nurses' Institution has dropped private nursing, there should be scope for reliable trained nurses for private work in Torquay.

### THE DURATION OF A DISTRICT NURSE'S VISIT.

At the annual meeting of the Chester and District Nursing Association, the Mayor, who is himself a doctor, in moving the adoption of the report, said he was surprised to find that so many visits had been paid—the number in the year was 21,046, there being a superintendent and five nurses—"because the visit of a nurse was much more prolonged than that of a doctor." This, as the Bishop of Chester pointed out in a subsequent speech, was emphatic testimony from an expert of the vast amount of time devoted to people by district nurses, as well as care and sympathy. Of course there are visits and visits. Sometimes the visit of the nurse is properly limited to an inquiry whether her services are required any further, but at others she may find it desirable to spend an hour with the patient. It is quite certain that it would never do for the district nurse to keep her eye on the clock, or regulate the number of visits strictly by the number of hours in the day.

### ANOTHER NURSE WANTED AT ABERAVON.

According to the newspaper report of the annual meeting of the Aberavon and Port Talbot Public Nursing Association, the attendance was "meagre but influential." We hope that the influential people who were present will lay to heart the appeal which was made to them to increase the income of the Association sufficiently to enable it to engage a second fully-trained nurse. At present the assistant nurse is not fully trained, and until the salaries of two fully-trained nurses can be guaranteed, the committee cannot be blamed for not entertaining the idea, especially as they have found the maternity nurse useful. But with 6,246 ordinary and 171 casual visits, or an increase of 2,985 on 1901, paid last year, it is obvious that there is abundant work for two nurses of experience in general and district work.

### ECONOMY AT STAFFORD.

It cannot be complained that the Stafford District Nurse Society waste money on superfluities. The total expenditure last year was £181, and only £5 of this amount was expended on the management. There is, however, no attempt to stint the two nurses who received £121 last year in salaries, and £44 for lodgings. In accordance with a recommendation from the Queen's Institute, the committee raised the allowance to their nurses from 10s. to 11s. 3d. per head per week. This recommendation was prompted by the advance in the price of coal and provisions, and it could not, of course, be disregarded. We notice with regret that only a few churches in Stafford assist the Society, and commend the proposal of one of the speakers at the annual meeting, who suggested that the committee should send a letter to each incumbent asking for a collection.

### PROGRESS AT WELSHPOOL.

From both points of view the Welshpool people may be congratulated upon the position of the Victoria Nursing Institute. The report for 1902, which was adopted at the annual meeting of subscribers, shows that a balance of £6 5s. on the wrong side has been converted into a balance of £30 4s. 9d. on the right side, and in addition to this marked improvement the handsome sum of £185, which was obtained by a sale of work and rummage sale, has been added to the capital account. The record of the nurses attests the increasing usefulness of the organisation, the number of visits paid being 2,729, and the sums given by patients themselves or their friends for benefits received amounting to £29 13s. 6d. Miss Howell, who five years ago asked the public to have a great deal of faith in the Institute, is now able to say that it stands upon its own merits.

### SHORT ITEMS.

The King has bestowed the decoration of the Royal Red Cross on Lady Macdonald in recognition of her services in tending the sick and wounded soldiers during the defence of the Legations at Pekin in 1900. Lady Macdonald is the wife of our former minister to the Emperor of China.—The s.s. *Lake Manitoba*, which arrived at Southampton last week, had on board Nursing Sister Shaw-Hillier of the Army Nursing Service Reserve.—The third annual drawing-room sale of work on behalf of the Peckham Nursing Institute will be held during the second week in June. It is hoped that a nurses' home may be eventually established by means of these sales.—The Incorporated Society of Trained Masseuses will hold an examination on Tuesday and Wednesday, March 17th and 18th. Candidates wishing to enter for it must apply to the Secretary at once.—Miss Mary Coyne, who has been appointed charge nurse at Aston Union Infirmary, was trained at Prescot Union Infirmary.

### The Nursing Outlook.

"From magnanimity, all fear above;
From nobler recompense, above applause;
Which owes to man's short outlook all its charm."

### POST-GRADUATE TEACHING.

WHEN a nurse leaves her training school after three years' rigorous discipline in the methods of that hospital, she is generally a very dogmatic and self-assured person. That is only human nature; you may notice the same thing in the young minister fresh from a theological college, or the assistant teacher just let loose from a training college. They have yet to learn that truth Jowett put so quaintly—"We are all of us liable to error, even the youngest of us!" A little knowledge of the world, and the variety of methods, soon teaches that toleration and philosophy which comes from experience, and so you get the elderly minister turning out a very kindly man, in no hurry to judge; you get the teacher learning to leave the children much more to nature, and you get the nurse ready to acknowledge that she does not know everything, and often eager to be kept in touch with the new developments and knowledge which affect her work.

Now, what has been done in the past in the way of post-graduate teaching for nurses? So far as we know, only the lectures at the Nurses' Club in Buckingham Street and at the Royal British Nurses' Association; and a post-graduate course open to all nurses at the Orthopædic Hospital. The training schools have practically said to the nurse who has taken her certificate and left: "We have done with you; you have no more claim on us, we can teach you no more!" In fairness to the London Hospital, which always has a high nursing ideal, we must state that the nurse who remains on the private staff in connection with the hospital is always taken back into the wards and allowed to attend lectures between cases. But the majority of nurses who have finished their training want a little freedom from hospital restraint, a little time between cases to rest and develop the mental and spiritual life, and is the hospital right in cutting these children entirely off from further technical teaching? We think not; we think that every hospital which is a big training school should feel that its reputation is in some degree in the hands of the nurses it sends forth into the world, and should try to keep a hold on those nurses and help to keep them in the front rank of their profession. Nurses are most loyal, as a rule, to their *alma mater*, as the present "Leagues" in connection with certain schools show. But the hospital ought also to be loyal to its old probationers. We do not believe there would be any difficulty in getting medical men or sisters to give lectures or demonstrations to old nurses, if only some-one would take the trouble to organise the matter; if only the matrons and the hospital committees could be interested in post-graduate teaching. But the large hospitals are always so busy striving to keep up with the times, and to secure the necessary funds, that it is only as the result of some self-sacrifice that this can be looked for. "Our duty is first to the patients; we have quite enough to do without furbishing up old-fashioned nurses," said the member of a hospital committee the other day. But the duty of our hospitals is to the public as a whole, and it is in the interests of the public *as a whole* that we plead for this effort to keep the private nurse in touch with new methods. It was part of the scheme of the Nurses' Co-operation to have lectures and post-graduate teaching for the nurses, but the purely nurses' parts of the co-operation have all been allowed to fall into abeyance. True, the need of such teaching is admitted by the fact that a surgeon wrote, and presented to all the nurses, a pamphlet on "Preparations for Operations in Private Houses." But one pamphlet in twelve years is a little inadequate. It somehow reminds us of the story of the horse that was trained to live on one straw a day—and of the fate of that horse! Seriously, we would ask the training schools before next autumn comes along to try if they could not organise some lectures and demonstrations for graduate nurses. Are old nurses to learn all the new theories on light and air treatment by being pitchforked into such cases? That is rather rough on the patients, for the nurse at a private case has not always a sister to supervise her work, or a house-physician to turn to in moments of danger or in case of mistake. And in the new operating methods irreverently described as "kid-glove" or "knife-and-fork," how is the old-fashioned nurse at the critical moment to recognise an instrument the surgeon wants when she has never seen such instruments before? We are certain that the medical profession are ready to teach, we are certain that the nurses are ready to learn; it remains only to plead with the training-school authorities to take up this one more duty towards the public who support the hospitals, and to arrange for the post-graduate teaching of old probationers.

If they refuse to afford facilities which are so definitely needed, an attempt should be made to induce the Poor Law infirmaries, members of the medical profession who have the nurses' interests and their own at heart, and some of the smaller hospitals to organise a thorough system of post-graduate instruction. We cannot think that the large training schools will, however, allow such an arrangement to come to pass. Nowadays, when the necessity of progressive knowledge on the part of nurses is admitted, surely those who have worked hard in the wards of the hospitals have a right to look to these institutions to provide them with opportunities to keep themselves efficient and equal to the demands of modern medical requirements.

### Lectures on Medical and Surgical Nursing.

By JOHN HOPKINS, F.R.C.S., Medical Superintendent of the Central London Sick Asylum District Asylums, Cleveland Street, W., and Hendon, N.W.

### LECTURE II.—INFLAMMATION.

WHEN microbes find their way into the tissues or on to the mucous membrane of the throat (more especially) where they can lodge and grow, they irritate the parts around. Living and growing, they absorb the juices they happen to lie in, and excrete their own waste products, which, being fluid, pass into the coats of the capillary blood-vessels and veins. Here, coming in contact with the white corpuscles of the blood, these at once adhere to the inside of the vessel, and presently pass through its coat, escaping from the veins and capillaries into the surrounding tissues, where they find themselves in the presence of the microbes. A battle at once begins between them. Each party is said to secrete something inimical to the other, with the result that both microbes and blood-cells are slaughtered, sometimes in great numbers. Others say that the white blood-cells (or leucocytes) do not secrete anything hurtful to the microbes, but eat them up and destroy them inside their bodies; and that this power of destruction on the part of the leucocytes is only reinforced when one of them dies, for that then it decomposes and there escapes from it a fluid that both neutralises the toxic matter secreted by the microbe and destroys the microbe itself.

At the same time that leucocytes issue from the veins and capillaries, there escapes a fluid part of the blood, and both together distend the part, which is thus swollen. At the same time the blood-vessels dilate to allow the arrival of a greater number of white blood corpuscles, and often they dilate so wide that the beat of the heart can be felt in the inflamed part, and it is then said to throb. This larger flow of blood causes redness, as the red colour of the blood shows through the skin. It also causes the sensation of heat and the part also feels hotter when the hand is laid upon it; but it is never hotter than the blood itself, which has the same temperature in the inflamed part as it has elsewhere. At the same time the nerve endings are compressed and become over-sensitive, so that pain is felt. Thus are accounted for the four classical signs of inflammation, redness, swelling, heat, and pain.

What happens afterwards depends upon the result of the contest. If the leucocytes win, we find a solid wall built round the nest of microbes, formed from the fluid that has escaped from the blood-vessels, which has coagulated; and the microbes together with leucocytes are enclosed in a sack which has been formed by the tissues being separated and thrust aside or absorbed. This sack is called an abscess, and the leucocytes together with the fluid in which they float constitute the matter or pus that is contained in the abscess. This pus acts as a foreign body which is not allowed to remain in the part it occupies, so by a constant addition of white blood cells to it, the pus grows in quantity and pushing its way along the path that offers least resistance to it, the abscess reaches the surface of the body, where it points and bursts. Nature may be greatly aided in this, for a cut made into the abscess with a knife at once lets out all offending matter, and then the blood-vessels return to their usual size, the swelling disappears, the lymph that had coagulated is absorbed, and the part returns to its normal state, presenting simply the scar that marks the site of the combat. That is the most fortunate result for the patient. If the microbes win the battle, they or their excretions gain access to the blood, and are carried all over the body, causing sapræmia, septicæmia and pyæmia. The first of these three is poisoning by the excreta of the microbes, the second is the poisoning by the microbes themselves, and the third is where, in addition to the former two, there is the formation of fresh abscesses in different parts of the body. Should the microbes fail to get into the circulation they may still succeed in eluding the leucocytes in the tissues for some time, and, by spreading, cause extensive inflammation of the skin and cellular tissues that may result in the formation of numerous abscesses round about, or in sloughing of the cellular tissues, or even gangrene of the skin.

The result of a contest between microbes and leucocytes, depends upon the strength and number of the microbes, and the power of combat possessed by the leucocytes. Persons already diseased, especially those with acute fevers, and those who have ruined their health by excessive drinking, show little power of resistance to the invasion of microbes. In such people harmless looking scales and scabs, presenting little or no signs of inflammation around them, may have beneath them collections of pus from which absorption of toxic matter is taking place and producing the gravest symptoms. So it is a golden rule, especially in an infirmary where are so many patients of the kind, never to permit any scabs or scales to remain. Always expose the raw surface and wash it well with antiseptic lotion. Erysipelas, sloughing cellulitis, phagedæna, and gangrene of the skin are especially to be feared in those whose constitutions are so weakened by disease that the inflammation around a nest of microbes is weak and does not lead to the formation of a wall of coagulated lymph around the bacteria.

*Erysipelas* is a spreading inflammation of the skin, which often attacks the face, but may invade any part. It is due to a micrococcus which grows and spreads in the capillary lymphatic vessels of the skin. Erysipelas, when it attacks the face, causes redness and swelling, the formation of vesicles and mattery points which are the mouths of the sebaceous gland-ducts filled with pus. Sloughing of the cellular tissues of the orbit sometimes occurs and is generally fatal.

*Lymphangitis* is an inflammation of the lymphatic vessels, and is recognised by red lines running up a limb.

*Lymphadenitis* is inflammation of the lymphatic glands. The bacteria are carried by the lymph vessels to the nearest glands, and wherever they are carried they set up inflammation.

*Cellulitis* is inflammation of the cellular tissue beneath the skin. If intense this may lead to sloughing of the tissue; that is, it dies, and when it comes away it is as a matted grey mass of fibres.

*Gangrene* of the skin sometimes accompanies the former when it becomes dark brown or black from changes in the blood that is stagnated in it. All the foregoing inflammations often occur in one and the same case, and they lead frequently to blood poisoning. When the inflammation leads to abscesses, as it fortunately often does, we know that the process of spreading has been checked wherever an abscesss forms. At first these abscesses are acute; but later in the case they are often subacute and cause little pain, so that the patient gives no warning of their presence.

In the nursing of erysipelas my advice has always been to allow no scabbing and to keep the part clean and free from any powder; also to open all mattery points twice daily, as well as the blisters that form, and to keep the part covered with a moist antiseptic dressing, making a mask for the face. But there are other methods of treatment which you may have to carry out in your career as nurses. In applying fomentations do not put them on too hot, lest the vitality of the part suffers as well as the nerves of the patient, and let them thoroughly enclose the part where that is possible, so that they will retain both their heat and moisture. Keep an eye upon the part in later stages so as to detect any soft spot, easily felt by the tip of the finger being rubbed lightly over the skin. These soft spots, generally painless, are often numerous and keep forming for days sometimes. They are abscesses, the presence of which is not always indicated even by a rise of temperature.

### After Fifty: Or Poor Law Nurses and Pensions.

By Miss WILSON, Treasurer of the Workhouse Infirmary Nursing Association; Member of the Midwives' Board.

(*Concluded from page* 278.)

### Poor Law Officers' Superannuation Act.

Now to come to the remaining plan by which Poor Law nurses are expected to provide for the future, *i.e.* the Poor Law Officers' Superannuation Act, 1896. I may frankly say at once that I actively opposed the inclusion of nurses under this Act, and that, supported by a consensus of expert opinion, and with the active help of Mr. Boulnois, M.P., Dr. Elwin Harris, and others, I exerted myself to obtain their exclusion for this particular class of workers with a hope that a just provision might be made for them later on. When this hope was rendered impossible through the rapid passing of the amending Bill in its last stages, owing to the energy of the promoters, an amending Act (1897) had to be accepted, which gave to nurses the power to contract out of the Act. While accepting this as a modicum of justice, I still felt that the question was wholly unsettled. The inclusion of nurses was unfair to a class whose claims under the Poor Law should have received special consideration, and whose conditions of service are in no way suited to the provisions of the Act.

### Drawbacks for Nurses.

The following extract from a matron's letter expresses the views on this question which are shared by many who have the nursing service of the Poor Law at heart:—"There are only four nurses here out of a staff of 93 who have joined the Poor Law Officers' Superannuation Fund. My observations are to the effect than unless the Poor Law Superannuation Fund were made more elastic there is no inducement for any nurse unless she be convinced that she is a confirmed spinster to join it. Had the Local Government Board inaugurated a scheme for the Poor Law Service offering the advantages of the Royal National Pension Fund, doubtless more nurses would join, but seeing that a subscriber to the Poor Law Superannuation Fund must be incapacitated by infirmity or old age before she can enjoy the fruits of her saving, and that in the case of death she cannot devise her savings to her friends, I think she needs the conviction (which can only be inspired when she feels old age is approaching) that she cannot vary her position to her betterment before she subscribes to such a fund. Another drawback to this superannuation fund is that it is held payable at the pleasure of a committee the components of which are constantly changing to such an extent that should a matron hold her post, say for ten years, she might then be working under a committee quite different from the committee which elected her, and which might, if so minded, make the continued holding of her office impossible, and the consequent receipt of her superannuation subscriptions an uncertainty."

How can a fund, formed with a special view to the position of workers who remain in one employment until their working days are ended, be made to apply to nurses who at 50 are often not "fit" in the best sense of the term for responsible ward duty? Some Poor Law nurses may and do work till 55 and later, but is it always with advantage to the patients, to the general well-being of the infirmary, to the improvement or training of younger nurses I think the honest answer must be No. Now that the call of efficiency is being heard in the land, can we in fairness to the care of the sick and the training of the young, to the spirit of energy that is in itself the best expression of mature power, say that a solution of this important question is provided by an Act which lays down as a first condition that no one shall retire on pension till the age of 60, unless incapacitated in mind or body?

### A Workable Pension Scheme Required.

Poor Law nursing is not popular for many reasons. Indeed, it is not too much to say that had the Act remained compulsory upon nurses as upon other officers, the applications for nurses' posts under the Poor Law, inadequate as they now are, would almost have ceased altogether. The Amending Act saved this catastrophe, but it did not furnish a solution of the difficulty. If hospitals do not offer better salaries they do offer more interesting cases, more varied experience, the stimulus of keeping in touch with the fresh developments of research, and of watching the results of new methods of practice above all, they offer and confer kudos—just as important a factor in a nurse's working life as in that of a soldier, and one which we can never leave out of sight while human nature remains what it is. The self-devoted nurse may often accept the less interesting field of Poor Law as her life work. No one can realise this fact from long experience with more gratitude than I do; but one must not reckon on the exceptions in human nature; and while there are Red Crosses for the army sisters and applause for the distinguished private nurses who are brought in the course of their duties to attend on the great ones of the earth, I ask for the Poor Law nurse, whose work is generally laborious, often thankless, and always little known, that she should be ranked as other civil servants are, who, while content to do their duty quietly in the background, are relieved of anxieties as to their future. I ask that, apart from the Board of Guardians that sees only her later work and is unable to appreciate her earlier energies, the Poor Law nurse should, if faithful in her high work, be entitled to a definite pension for her old age, absolutely, and without reference to individual opinions or circumstances. The condition under the Army Nursing Service is a pension after 15 years' work. The Poor Law Nursing Service is no less important, no less worthy, and if a retiring pension were a nurse's due at the age of 50, or at the outside limit 55, she would frequently have earned it by double the length of service required for the Army pension. Let there be some sort of equality between the two services if nursing in our State hospitals is to progress, and to maintain a proper standard of efficiency.

The situation is undoubtedly grave, and the Poor Law Department would act wisely to offer greater attraction to its nurses without delay. Already the supply is inadequate; private, district, and colonial nursing all attract nurses by better pay, greater freedom from red tape, or by better surroundings and more interesting work. If the President of the Local Government Board can see his way to devising a fair scheme for nurses, absolutely apart from the Superannua-

### AFTER FIFTY: OR POOR LAW NURSES AND PENSIONS—*Continued.*

tion Act, he will do a difficult perhaps, but most useful, service, not only to the sick but to the community at large. We desire to see action taken which will help to keep nurses attached to the Poor Law service as in part their life work, and to which they will give of their best, without anxiety for the future. We have seen that it is difficult for nurses to secure this end by their unaided efforts, and it can hardly be doubted that the solution rests largely with the Local Government Board in framing a workable scheme which would be just and acceptable to Guardians and nurses.

### Departmental Report and Pensions.

This paper was written before the publication of the Report of the Departmental Committee on the Nursing of the Sick Poor in Workhouses. But I do not think that the contents of the report suggest any alteration or modification in what I have written. My views are indeed strongly confirmed under the heading of "increase of comfort," paragraph 58 (5) of the Report, which recommends that—

(5) Guardians should be encouraged to pay part of the premiums towards annuities for their nurses.

The committee understand that nurses have very generally taken advantage of the Poor Law Officers' Superannuation Act of 1897 to contract out of the similarly named Act passed in 1896. This is apparently chiefly due to the fact that the minimum age at which a pension is obtainable under the Act is too high to be of any general benefit to nurses, having regard to the arduous and exhausting nature of their duties.

It is felt that the certainty of a pension at a reasonably early age would add very materially to the attractiveness of the Poor Law Nursing Service, and the committee urge very strongly that Guardians should, in all cases of nurses who have contracted out of the Act of 1896, contribute towards the premiums of the nurse's annuity on some scheme similar to that obtaining in some of the London hospitals. If necessary, special powers should be given to the Guardians for this purpose.

### Appointments.

[No charge is made for announcements under this head, and we are always glad to receive, and publish, appointments. But it is essential that in all cases the school of training should be given.]

Belfast Union Infirmary.—Miss Sarah Ward has been appointed superintendent nurse. She was trained at Brownlow Hill Infirmary, Liverpool, where she was afterwards night superintendent. She has also been superintendent at Sculcoats Union Infirmary.

Chelmsford Union Infirmary.—Miss Annie Mary Dell has been appointed superintendent nurse. She was trained at Salford Union Infirmary, and has since been charge nurse at West Ham Infirmary for three years.

Christchurch Union Infirmary. — Miss Elizabeth Allen has been appointed superintendent nurse. She was trained at Stafford General Infirmary and Whitechapel Union Infirmary. She has since been superintendent nurse at Stroud Union Infirmary, at Billericay Union Infirmary, at Wolstanton and Burslem Union Infirmary, and at Prestwich Union.

East London Children's Hospital, Shadwell.—Miss Patience Shearman has been appointed staff nurse for the Convalescent Home at Bognor. She was trained at Her Majesty's Hospital, Stepney Causeway, E.

East Parochial Hospital, Dundee.—Miss Elizabeth Anna Smith has been appointed charge nurse. She was trained at the Royal Hospital Donnybrook, Dublin, and at "The City of Glasgow" Hospital, Belvidere.

East Suffolk Hospital, Ipswich. — Miss Emilie Barnecut has been appointed night sister. She was trained at the Royal Albert Hospital, Devonport.

East Suffolk Hospital, Ipswich. — Miss Margaret Lippiatt has been appointed sister of the women's medical ward. She was trained at the Bristol General Hospital, subsequently obtaining the Apothecaries' Hall qualification for dispensing. For the last two and a-half years she has served with the nursing staff of the Army in South Africa as a member of the Army Nursing Service Reserve.

Grimsby and District Hospital.—Miss Marion Barwell has been appointed sister. She was trained at the General Infirmary, Leeds, and has since been sister at Cardiff General Infirmary. She is a member of the Army Nursing Service Reserve, and for two and a half years has been nursing in South Africa and at home stations.

Hackney Union Infirmary.—Miss Kate L. Nightingale has been appointed charge nurse. She was trained at the Brentford Union Infirmary, where she has since been staff nurse.

Ham Green Hospital, Bristol.—Miss Lilian Shipway has been appointed charge nurse. She was trained at the Lambeth Infirmary, was for upwards of five years on the private staff of the Trained Nurses' Institution, Leicester, and has lately been day sister at the Infirmary, Kingston-on-Thames.

Preston Union Infirmary.—Miss S. A. Brierley and Miss Clara Edith Armitage have been appointed charge nurses. Miss Brierley was trained at Queen Charlotte's Hospital, London. Miss Armitage was trained at Rochford Union Infirmary, Essex, and has since been assistant nurse at Swindon and Highworth Union Infirmary, and charge nurse at Milton Union Infirmary, Kent.

Queen's Jubilee Hospital, Earl's Court.—Miss Edith Meyers has been appointed matron. She was trained at St. Thomas's Hospital, and has since been sister at St. Monica's Hospital, Brondesbury, London.

Stockport Infirmary.—Miss S. Varley has been appointed night sister. She was trained at the London Fever and Royal Portsmouth Hospital. She has since acted as holiday sister, night sister, *pro tem*, and out-patient sister at the Royal Portsmouth Hospital.

### Death in Our Ranks.

The death, from appendicitis, took place on Saturday last, at St. George's Hospital, of Miss Mary Stephenson aged 27, a member of the Wigmore Nurses' Institution, 59 Weymouth Street, W. Miss Stephenson, who had been ill only four days, was nursing a patient when she first complained of great pain. An operation was performed immediately she was admitted to the hospital, but it was too late.

The death of Miss Kathleen Morney, charge nurse at Calverley Hospital, Thornbury, at the age of 29, occurred on Wednesday, last week, after two months' suffering. Miss Morney was trained at Limerick Infirmary, and had been on the staff of the Harrogate and Wakefield Trained Nurses' Home before she was appointed to Calverley Hospital where she was very popular.

### The Nurses' Co-operation.

WE have received the twelfth annual report of this Society. The committee rightly describe their meagre report as "customary." However regrettable, it is certainly customary for the committee to give neither facts nor full information which will enable the nurse-members of the Co-operation or anybody else to fully appreciate the actual position of the Co-operation, its progress, or its retrogression. Like the majority of the committee of the Zoological Society, the majority of the committee of the Nurses' Co-operation appear to make it their aim to hamper progress and to hold possession of other people's property, regardless in large measure of the fact, that, after all, both these societies concern the public, and cannot be permitted to gradually bleed to death for want of enterprise and business methods in the conduct of their affairs. The majority of the committee of the Zoological Society have brought the gardens to a miserable pass of inefficiency. The present majority of the committee of the Nurses' Co-operation, if left alone, seem fated to steer that enterprise on to the rocks of public discredit, without any compensating advantages. Further than this what is to be said of a finance committee which omits from its report all allusion to the circumstance that there is a serious leakage in the business? The able firm of auditors, Messrs. Craggs, Turketine and Company, of course show in the income and expenditure account for the year 1902 that there has again been a loss of upwards of £255 on the nurses' home and club. The expenses connected with the Home were, in round figures, £2,013, and the receipts £1,758, showing a loss on the year of £255. Why should the nurses who join the Co-operation be asked to pay this fine out of their hard-earned moneys year by year? And if they have to pay it, what can be said of a finance committee which omits all mention of the circumstance from its report (*vide* page 13 of the twelfth annual report of The Nurses' Co-operation)?

### Everybody's Opinion.

**[Correspondence on all subjects is invited, but we cannot in any way be responsible for the opinions expressed by our correspondents. No communication can be entertained if the name and address of the correspondent are not given as a guarantee of good faith, but not necessarily for publication. All correspondents should write on one side of the paper only.]**

### GOLD WATCHES FOR SMALL-POX NURSES.

"VACCINATION" writes: It would be interesting to know whether the Romford Guardians are defraying the cost of the gold watches for their courageous nurses out of their private purse; if not, will such an item of expenditure be allowed by the auditor? Nurses as a rule are willing to accept considerable inconvenience and isolation in order to gain experience of small-pox, and when one remembers that of all the zymotic diseases it is the only one from which we can be sure of immunity, to make heroines of those whose duty has brought them into contact with small-pox appears to be a rather exaggerated and extravagant doctrine.

### THE ASYLUM NURSE

"X. L." writes: In THE HOSPITAL *Nursing Section* of February 7th I read the article, "The Asylum Nurse," with a good deal of admiration. But it is not a true picture to say that patients in asylums have for the most part "the vacant stares, the restless rolling eyes, the crouched, huddled form of the melancholiac, the ceaseless, senseless babble of the childish adult, the wild and noisy maniac." There are, for instance, many melancholiacs with no crouch, no inclination to huddle. There are many people in asylums with no outward or visible sign of madness. Many men and women, and alas! girls, good looking, alert, and bright, but nevertheless with the dreadful taint of madness somewhere. Another remark needs comment. Writing of when an asylum nurse is called in to a private mental case—you say:—"But, on the other hand, when an asylum nurse has been called in to a private *mental* case, *the* (the italics are mine) *asylum methods have been too rough for the relations to stand.*" If the asylum methods are too rough for the relations to stand in their homes why do these relations in thousands send their unhappy private mental cases in thousands to these asylums? Why does the great voice of the British Public not say as one man these methods must cease. I have no wish to throw stones, but if in a hospital for the "Sick and Injured" fifty-two helpless patients had been burned to death—say at St. Thomas's, "Guy's," any hospital—what, I repeat, would have been said, if no nurse nor doctor had perished? I have had experience in four asylums and know what can be done with lunatics, and I still say, as thousands in England are saying, it would be better for the credit of Colney Hatch if those who perished had not all been of unsound mind.

### NURSING BY ORDERLIES.

"RESERVE SISTER" writes: The letter from "Private A. J. Power" exactly bears out what I have said as to the sister's position in the Army. She is responsible for, that is to say she has to see that the orderlies perform, "the nursing of the patients, and that the food, medicine, stimulant, etc., ordered are administered to them. The orderly is mistaken in thinking that the sister is expected to do all the nursing and fetch the food, stimulants and medicines for the patients herself! If he will stop to think he will see that as she is "in charge" of several wards very often this would be a physical impossibility. She has to supervise the performance of these duties by the orderlies. But he says "men will not be controlled by women." Also "she is the weaker vessel which has to be guided by man." Unfortunately for "Private A. J. Power" this is not the view of the War Office on the subject. It does not look on the sister as the weaker vessel to be guided by man in the shape of the orderly! but does expect the sister to control the orderlies, especially now that the ward master exists no longer, to instruct them in nursing and to give them lectures. That is where the shoe pinches; and some orderlies try to show their fancied superiority to the "weaker vessel" by persistently smoking when she is present or by remaining full length on a bed in the ward when she enters it. But I must say that the majority of orderlies I have worked with were courteous, willing, and hardworking, especially the regimental ones whom your correspondent terms the rougher element. I am only sorry he has not been equally fortunate in his experience of Reserve Sisters. I was at four different hospitals in South Africa during nearly three years. The night sisters always made a round of the wards under their charge immediately on going on duty at 9 P.M. and every two hours after till 7.30 A.M.; visiting bad cases every hour or oftener. Their duties were not confined to giving medicines and stimulants. They supervised the nursing, watched the patients' condition, sent when necessary for the medical officer and often did spongings, bed-making, dressings, etc., and applied poultices and fomentations.

### "The Hospital" Convalescent Fund.

THE Hon. Secretary begs to acknowledge with thanks the receipt of 2s. 6d. received from the Editor of THE HOSPITAL, who has kindly undertaken to hand over to this Fund all the fees received from correspondents requiring immediate answers to their queries in the "Travel" column.

### Where to Go.

MONDAY, TUESDAY, WEDNESDAY, MARCH 16TH, 17TH, 18TH.—Sale at the London Shoe Company's Branch, 21 and 22 Sloane Street, S.W.

### The Nurses' Bookshelf.

THE NURSE IN HOT CLIMATES. By EDWARD HENDERSON, M.D., F.R.C.S.Edin. Revised and Reprinted from THE HOSPITAL Journal. (The Scientific Press, Ltd. 1s. net.)

A VERY handy little book has been compiled from a series of papers which have appeared in our columns, and which will undoubtedly form a very useful guide to nurses intending to go abroad, and especially to tropical climates. All the directions are very clear, and the nursing details most carefully given. The bamboo settee described on page 20 to be used as a bath in cases of hyperpyrexia is worthy of note, and might be usefully imitated in our own hospitals. "Washing the hands in turpentine," page 44, is rather a severe measure for disinfecting them, as its use renders the skin extremely tender, and roughens it so much, thereby affording increased risks of abrasions. To discourage the use of the bed-pan in midwifery cases, page 37, is perhaps a little to be deprecated. With the exception of these minor criticisms, the "Nurse in Hot Climates" is a most concise and valuable little handbook.

A HANDBOOK FOR NURSES. By J. K. WATSON, M.D.Edin., M.B., C.M. Second and Revised Edition (Eighth thousand). (The Scientific Press, Limited.)

A SECOND edition of this compendious work should be welcomed by the nursing world. In an excellent preface the author sets forth plainly the objects and special nature of his text-book, and he has succeeded in maintaining throughout a fair balance between the theoretical and practical work and knowledge of a nurse. The nursing directions are clearly and sensibly detailed, and the proper duties of a nurse as distinguished from the medical man are wisely set forth, especially in the chapter on "Midwifery Nursing." In the preface to the second edition the author courts any further suggestions, and we would suggest, in the chapter on bandaging, more detailed directions on, with illustrations of, the uses to which the triangular bandage may be put, *e.g.*, as taught by the St. John's Ambulance Society. As a minor criticism in this connection the method of applying a sling to the elbow and arm, depicted on p. 218, fig. 55, does not strike us as so effective or neat in appearance as the usual method. A good index and table of contents and two useful appendices complete this thoroughly up-to-date nursing text-book.

### Presentations.

HOSPITAL FOR WOMEN, LIVERPOOL.—Great regret was felt at the Hospital for Women, Shaw Street, Liverpool, on the resignation of Miss Evelyn G. Tobin upon the occasion of her marriage with Dr. J. Abercrombie, honorary physician to Charing Cross Hospital. Although the stay of Miss Tobin at Shaw Street was short, she had by her bright, genial manner endeared herself to all who came in contact with her. On leaving the hospital she was presented by the honorary staff with a handsome old English salver of the time of George III., by the nursing staff an old English silver cream jug, and the maids with a copper crumb brush and tray.

MERTHYR VALE DISTRICT NURSING ASSOCIATION.—Miss Lucy Hill, who has been Queen's nurse at Merthyr Vale for the last two years, and is taking up the duties of superintendent nurse of the Pontypridd and District Nursing Association, has been presented with a silver tea service, a handsome case of silver Apostle spoons and sugar tongs, and a writing case in Russian leather, as a token of esteem from the friends amongst whom she has worked.

### TRAVEL NOTES AND QUERIES.

CHANNEL ISLANDS (Spring Holiday).—We do not insert questions, but they are answered by the Travel Editor. April is a good month in that locality, though May is usually better. The return fare to Jersey is, first class, £2 8s., second class, £1 17s. 6d. This ticket will allow you to break the journey at Guernsey either going or returning, but not both. Hotel accommodation may be had for 5s. 6d. and 6s. per day. A week is rather a short time. Would you like to spend it between Jersey and Guernsey, or would you prefer Sark to Guernsey? Sark is wild and romantic: the "Coupée" is like nothing else in the world, but it is not so gay as the other places. It is possible to visit Sark in a day from Guernsey. Steamers go four times weekly—fare 2s. 6d.—and you are allowed from five to seven hours on the island, according to tide. Hotels at Jersey:—The Star, 6s.; The Weighbridge, 5s. 6d.; The Grasshopper, 6s.; The Temperance Hotel, at 31 Broad Street, 6s. 6d. At Guernsey:—The Crown, 5s. 6d.; and The Broughton, 6s. 6d. I think if economy be essential it would be well to remain only in one island, as your time is limited. Tell me if I can help you further.

HOLLAND IN SPRING (H. K. P.).—Write to me when you have settled exactly where to go after reading my articles. I should advise The Hague, Rotterdam, and Amsterdam, if pictures are your chief weakness. Yes, Holland is rather expensive compared to France and Germany, and the money exchange is not so favourable; but I can tell you of some reasonable quarters. Decide your points of residence and write to me again. I do not think you will be too late for the bulbs.

SWISS HOLIDAY (Yungfrau).—Very pleased to hear from you again. I think I can suggest the very place you want. Meirengen, between the Brunig Pass and the Lake of Brienz. Fare, second class return, £5 11s. From Lucerne you go over the Brunig to reach Meirengen. Accommodation may be had in June from 5 francs per day. There are beautiful walks all round. The Gorge of the Aare, the Gorge of the Alpbach, the Reichenbach Falls, the splendid walk to Rosenlavi Glacier, are all very easy excursions; and then being on the Brunig Mountain line is delightful; you can go up by train and walk back. Then the two lakes of Brienz and Thun are close and easily visited. Longer excursions may be made by the help of driving, and one can generally share a carriage with others. To the Grimsel Pass, for instance, is a most lovely walk. Tell me what you think of this, and how much you can spend each, and I will advise you as to hotels. I know of a personally conducted party going, semi-private and not undertaken for profit. Three weeks, starting the first week of June, cost about £10. It is, to my mind, the most perfect month in Switzerland: the flowers are then in perfection.

CORNWALL IN APRIL (E. M. A.).—You do not send a pseudonym, but I hope this will meet your eye. For the terms you mention it will be easy to get good lodgings. How would you like Tintagel? There is a large hotel there, but still there remain two homely little inns, "Wharncliff Arms" and "King Arthur's Arms" where you could have private rooms. It is a quiet place with magnificent coast. Then there is Bude Haven. Personally I prefer Tintagel to almost any spot in Cornwall, but Bude is gayer, and many much prefer Newquay, from which numerous excursions are to be made. Lodgings abound there. We do not keep a list, but if you went for a night to one of the hotels you could easily settle in rooms if you prefer to do so. I like best the side of Newquay away from the town and towards Porth. If you prefer the south coast, I think you would like Marazion (St. Michael's boarding house) from which you can visit St. Michael's Mount and all the magnificent coast round Land's End.

BELGIUM AT EASTER (River Thames).—Thank you for all the kind things you say. It helps me so much to know I have given pleasure, but the generality of readers "swallow and say nothing!" The articles you want the Manager will send you if you give him the dates and stamps for payment. There are six articles on Belgium as follows:—Bruges, September 2nd, 1899; Brussels, October 21st, 1899; Brussels, October 28th, 1899; Antwerp, August 3rd, 1901; The Musée Plantin, August 10th, 1901; and Mechlin, June 28th, 1902. Ask me any questions you like and I shall be pleased to answer, but I do not know what information you chiefly want.

TRAVEL EDITOR.

### Echoes from the Outside World.

### Movements of Royalty.

THE King held the first levée of the season at Buckingham Palace on Monday. He was in military uniform and, attended by all the great officers of State, entered the levée chamber shortly after midday. The ceremony only lasted three-quarters of an hour, and amongst those present were the Prince of Wales, Prince Christian, the Archbishop of Canterbury, Lord Roberts, the German, Russian, Spanish, French, Austrian, Turkish, and American Ambassadors. Also Governor Francis, of Missouri, President of the St. Louis Exposition, and the Hon. John Barrett, late Minister to Siam and now Special Commissioner from the St. Louis Exposition to Asia and Australia, were presented. The Earl of Arran was thrown from his horse just before the levée whilst doing duty with the Life Guards in the forecourt of the Palace. He, however, got back into the saddle and resumed duty, appearing none the worse for an accident which might have had disastrous results.

The King and Queen have expressed through Lord Knollys the satisfaction they derived from their visit to the model dwelling-houses at Millbank, but have suggested to the London County Council that the inmates should be provided with a larger number of cupboards.

### Close of Mr. Chamberlain's Tour.

MR CHAMBERLAIN made a considerable stay in Cape Colony. On Saturday he had an important interview with 150 delegates from the Dutch party at the Cape. The most prominent spokesman was Mr. Hofmeyr, and he was supported by Mr. Merriman and Mr. Sauer. At the conclusion of Mr. Hofmeyr's address, Mr. Chamberlain said that the statement he had just heard was "admirable in spirit and more calculated to make for that peace and good will which ought to prevail amongst all classes than anything that had been said or done up to the present." He accepted it, "as a most hopeful and happy augury for the future." Mr. and Mrs. Chamberlain spent Sunday at Simonstown. On Monday the Colonial Secretary received a deputation of loyal Dutchmen of Cape Colony; on Tuesday he was the chief guest at a farewell banquet in Cape Town; and on Wednesday he sailed from South Africa, exactly two months after he landed at Durban.

### The New Bishops.

NO fewer than four changes in the Episcopate were officially announced on Tuesday. The See of Winchester, vacated by the elevation of Dr. Randall Davidson to the Primacy, has been conferred upon Dr. Herbert Ryle, Bishop of Exeter since December, 1900; the See of Exeter, vacated by Dr. Ryle's translation, upon the Rev. Dr. Archibald Robertson, Principal of King's College, London; the See of St. Albans, rendered vacant by the death of Dr. Festing, upon Dr. Edgar Jacob, Bishop of Newcastle; and the See of Newcastle, vacated by the translation of Dr. Jacob, upon Dr. Arthur Lloyd, Bishop Suffragan of Thetford, who was vicar of Newcastle from 1892 to 1894. The new Bishop of Winchester is 47 next May; the new Bishop of Exeter not quite 50; the new Bishop of St. Albans is 59; and the new Bishop of Newcastle about 60. The whole of the four bishops are the sons of clergymen, Dr. Ryle being also the son of a bishop. The Bishop of Exeter, like the late Primate, has never been a parish priest.

### Signor Marconi on Wireless Telegraphy.

SPEAKING at the Savage Club on Saturday evening, Signor Marconi, who had an enthusiastic reception, said that his system of wireless telegraphy is now used on 25 trans-Atlantic steamers, and that it has established communication with stations on both sides of the Atlantic, which are not only valuable to passengers, but in some instances have been serviceable in insuring the safety of ships. These land stations could be spoken from a distance of 200 miles, but he confidently hoped to increase the distance very considerably shortly. Signor Marconi proceeded to acknowledge the great encouragement which he had received from the King. As early as 1898, he said, his Majesty, as Prince of Wales, had lent the Royal Yacht *Osborne* for three weeks at a time for his experiments. "As half a Britisher himself," he added that he should be sorry if the result of the policy of caution on the part of the British Government were that every Continental nation reaped the advantage of wireless communication before England; and he therefore hoped that their deliberations would come to a conclusion soon.

### The Ysaye-Busoni Concert.

THOSE who went to Queen's Hall last Friday afternoon had the pleasure of enjoying a great musical treat. The first item on the programme, Rubenstein's sonata in B minor, though treated by the two performers, Messrs Ysaye and Busoni, in thoroughly artistic style, is not capable of raising the enthusiasm of an audience to fever pitch, and many were the expressions of regret that the "Kreutzer" sonata, which was placed as a finale, had not been put earlier on the programme. Mr. Ysaye contributed three solos. He gave a transcription of some passages from "Parsifal" by Wilhelmj, followed by a delicious trifle from his own pen, entitled "Lointain passé," played as none but Ysaye could play it. The "Airs Russes" of Wieniawski which came last wrought up his hearers to such a state of delight that the violinist found it impossible to refuse an encore any longer. Busoni, too, was in his happiest mood, and his rendering of Schubert's "Wanderer" fantasia was not only masterly in the extreme, but replete with tender grace and poetic beauty. Madame Sobrino sang an air from Haydn's "Seasons," and then continued with three songs by Fritz Volbach, which were charmingly sung, and only suffered from an English translation being substituted instead of the original German words.

### Tolstoi's Play.

THE new play at His Majesty's Theatre is a play with a purpose and shows that Tolstoi as well as Tennyson believes that "Men may rise on stepping stones of their dead selves to higher things." "Resurrection" has been dramatised by Henry Bataille and Michael Morton, and consists of four acts and six scenes. Prince Nehludof in the prologue is being welcomed home by the villagers in truly Russian style. Here he renews acquaintance with Katusha, once an old playfellow and now a favoured domestic. The Prince makes love to her and the first wrong step is taken. Ten years later, Prince Nehludof is suddenly confronted again with Katusha in a court of justice, where, after a shameless life, she is accused of murder. He is stricken with remorse because he feels that it was his wrong-doing which first started her on her downward career. He determines to atone, breaks off his engagement with his fiancée, and endeavours to marry Katusha, who is now a sordid and brutalised human being. He follows her from prison to Siberia, where she goes as a convict, and there are some wonderful and striking scenes. Ultimately Katusha, who, too, has become regenerated, refuses to spoil the Prince's life by wedding him, and when he brings her a pardon, bids him return to Russia, whilst she will devote herself to alleviating the convicts' lot. Miss Lena Ashwell as Katusha proves her claim to be considered one of the strongest actresses of the English stage, and Mr. Tree is also admirable. There are nearly forty speaking characters in "Resurrection," and Miss Miriam Clements and Mr. Lionel Brough deserve special mention.

### For Reading to the Sick.

"THERE IS A SECRET PLACE OF REST."

There is a secret place of rest
God's saints alone may know;
Thou shalt not find it east nor west,
Though seeking to and fro.
A cell where Jesus is the door,
His love the only key:
Who enter will go out no more,
But there with Jesus be.

If thou hadst dwelt within that place,
Then would thine heart the while,
In vision of the Saviour's face,
Forget all other smile;
Forget the charm earth's waters had,
If once thy foot had trod
Beside the river that makes glad
The city of our God.

*From "The Inner Life."*

Our Lord's first recorded word is "Father"—"Wist ye not that I must be about My Father's business?" His first word from the Cross was "Father"—"Father, forgive them, for they know not what they do," so He ends as He began with "Father." It was a death, as well as a life, of perfect obedience. All the suffering was the obedience of a son! He is a loving son to the last, and He is not ashamed to call us brethren.

It is the same Father, then, Who watches over us, with the same tenderness of love. Do we always look up to God, in great suffering, with the same trustful confidence? Don't we have hard thoughts of God, austere thoughts? Don't we think of Him as an austere man?

When shall we learn to be trustful and thankful for everything that He sends us, even for suffering?

He is still thinking in the language of the Psalms: He takes the psalms and makes them personal, for He adds the word Father; and since He died with those words on His blessed lips, how many deathbeds have not these words sustained and comforted? "Father, into Thy hands I commend My spirit."—*Randolph.*

I know that Thou wilt never leave
The soul that trembles while it clings
To Thee; I know Thou wilt achieve
Its passage on Thine outspread wings.

I cannot see the golden gate
Unfolding yet to welcome me;
I cannot yet anticipate
The joy of heaven's jubilee.

But I will calmly watch and pray,
Until I hear my Saviour's Voice
Calling my happy soul away,
To see His glory, and rejoice.

*From "The Dove on the Cross."*

### Notes and Queries.

**The Editor is always willing to answer in this column, without any fee, all reasonable questions, as soon as possible.**

**But the following rules must be carefully observed:—**

**1. Every communication must be accompanied by the name and address of the writer.**

**2. The question must always bear upon nursing, directly or indirectly.**

**If an answer is required by letter a fee of half-a-crown must be enclosed with the note containing the inquiry, and we cannot undertake to forward letters addressed to correspondents making inquiries. It is therefore requested that our readers will not enclose either a stamp or a stamped envelope.**

*District Training.*

(160) Can you tell me if it is possible to get twelve months' training in a district nursing home, and to whom to apply for such training?—*Sister A. M.*

Two years' general training is required by the Queen Victoria's Jubilee Institute for Nurses (apply to the General Superintendent, St. Katharine's Precincts, Regent's Park, N.W., for all particulars), but the Affiliated Benefit Nursing Associations, 12 Buckingham Palace Road, S.W., trains for what are known as cottage nurses, and you might write to them. Also the Maternity Charity and District Nurses' Home, Howards Road, Plaistow, E., might help you.

*Abroad.*

(161) Will you kindly tell me if there is any English nursing institution in Vienna or elsewhere in Austria. I should be very grateful for any information about English nursing institutions abroad?—*Ethna.*

In no country have trained nurses such advantages as in England. On the Continent professional nursing is on an inferior basis, and the pay lower than at home, whilst the greater number of institutions providing English nurses are private. We have no particulars of any English hospitals or nursing institutes in Austria.

*Hospital Training.*

(162) Will you kindly tell me if there is any chance of getting into a hospital after passing the examination of the Apothecaries' Society as dispenser?—*L. F. T.*

Certainly, there is a chance of obtaining employment as dispenser in a hospital if you are qualified, but at present only very few women dispensers hold such appointments.

*Life Patient.*

(163) Will you kindly tell me of a public institution where a young man suffering from paralysis can be received for life for payment?—*K. C. W.*

You might apply to the National Hospital for the Paralysed and Epileptic, Queen's Square, Bloomsbury, W.C., for advice, also to the St. Peter's Home and Sisterhood, Mortimer Road, N.W., as the case might be eligible for one of its branch homes.

*Home.*

(164) Will you kindly tell me of any convalescent home in England where a lady recovering from an acute attack of mental hysteria could be received for 10s. weekly?—*M. E.*

Apply to the Secretary, the After-care Association for Poor Persons Discharged Recovered from Asylums for the Insane, Church House, Dean's Yard, Westminster.

*Massage.*

(165) I should be glad if you will kindly give me some particulars as to where I could receive lessons in massage, the average time it takes to become proficient, and the cost.—*Revrax.*

You can have lessons at the National Hospital for the Paralysed and Epileptic, Queen's Square, Bloomsbury, W.C.; the time occupied is from two weeks to three months, and the approximate cost is from £10 10s. to £40.

**Standard Nursing Manuals.**

"The Nursing Profession: How and Where to Train." 2s. net; 2s. 4d. post free.

"Nursing: Its Theory and Practice." (Revised Edition). 3s. 6d. post free.

"Surgical Ward Work and Nursing." (Revised Edition). 3s. 6d. net.; 3s. 10s. post free.

"Practical Handbook of Midwifery." (New Edition). 6s. net; 6s. 3d. post free.

"Notes on Pharmacy and Dispensing for Nurses." 1s. post free.

"Fevers and Infectious Diseases." 1s. post free.

"The Art of Massage." (New Edition). 6s. post free.

